# Supplementary material for: Phosphoproteome dynamics during mitotic exit in budding yeast
Source: EMBO J. 2018 Apr 12;37(10):e98745. doi: 10.15252/embj.201798745 (PMC5978319; doi:10.15252/embj.201798745)
Supplement: Supplementary file 1 — Expanded View Figures PDF [file EMBJ-37-e98745-s001.pdf]

Expanded View Figures

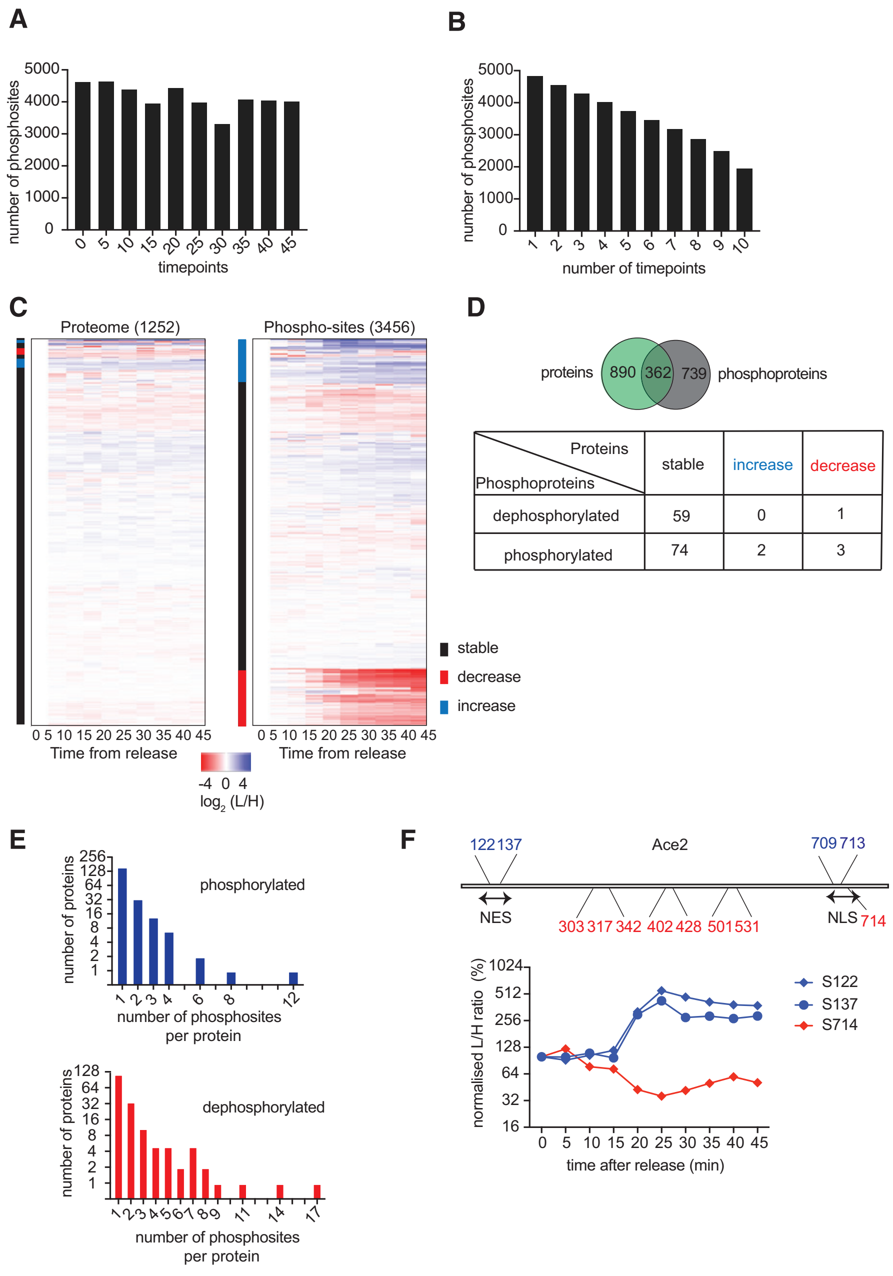

Figure EV1. Phosphoproteome and proteome changes during mitotic exit.

- A Number of phosphosites identified and quantified at each timepoint.
- B Cumulative number of phosphosites identified and quantified at least at the indicated number of timepoints.
- C Heatmap of abundance changes of the 3,456 phosphosites on the right and levels of 1,252 proteins on the left, during progression through mitosis. The L/H ratio in both cases was normalized to 100% in metaphase (0 min). Rows were ordered by unsupervised hierarchical clustering. Changes during mitotic exit were annotated as increase (blue), decrease (red), and no change (black).
- D Overlap between the proteins quantified in the proteome [non-enriched dataset (green)] and the phosphoproteins [phosphoenriched dataset (gray)]. The table contains a breakdown of protein abundance changes for proteins that harbor dephosphorylated or phosphorylated sites.
- E Distribution of the number of phosphosites per protein that were phosphorylated (blue) or dephosphorylated (red).
- F Schematic of Ace2, including the NES and NLS, as well as the phosphosites found to be dephosphorylated (red) or phosphorylated (blue), during mitotic exit. A profile plot of Ace2 phosphosites within the NES and NLS is shown.

 © 2018 The Authors

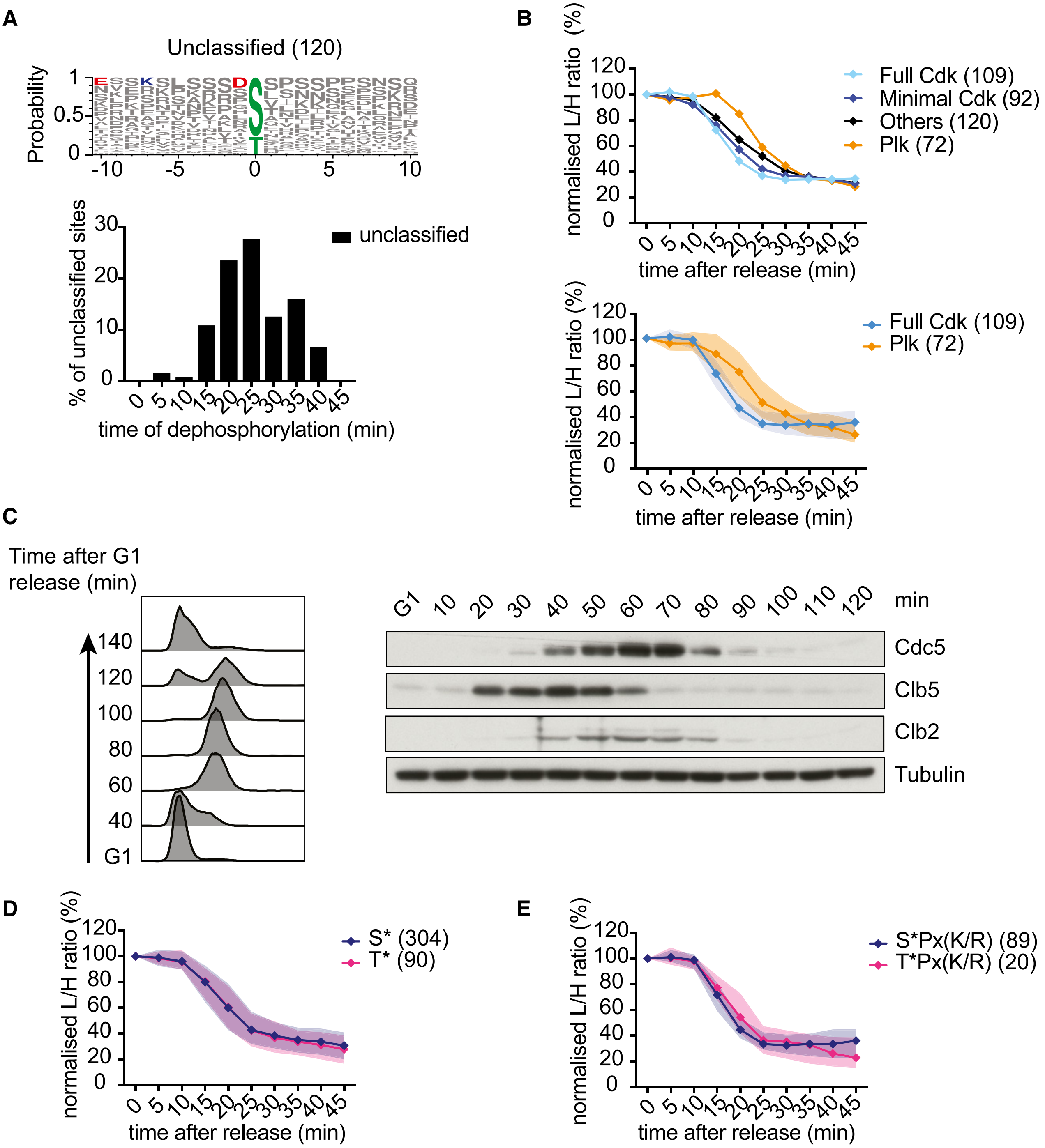

Figure EV2. Motif analysis of dephosphorylation during mitotic exit.

A Sequence logo of the 120 unclassified dephosphorylated sites that do not adhere to a Cdk or Polo consensus motif and the time distribution of their dephosphorylation during mitotic exit.

B Average intensity profile over time of phosphosites that adhere to the three indicated kinase consensus motifs. Median intensity profiles and interquartile range of the 109 Cdk and 72 Polo consensus sites are also presented.

C Abundance changes of Polo kinase and Clb5 and Clb2 cyclins during the cell cycle. FACS analysis of DNA content confirms α-factor-induced cell cycle arrest in G1, followed by synchronous progression through one cell cycle before re-arrest in the following G1. Cells expressed Cdc5 fused to an HA epitope tag for Western detection. Clb5 and Clb2 were detected using antibodies raised against these cyclins, tubulin served as a loading control.

D Median intensity profiles and interquartile range over time of all dephosphorylated serine and threonine sites.

E As (D), but showing dephosphorylated serine and threonine sites within full Cdk consensus motifs.

© 2018 The Authors

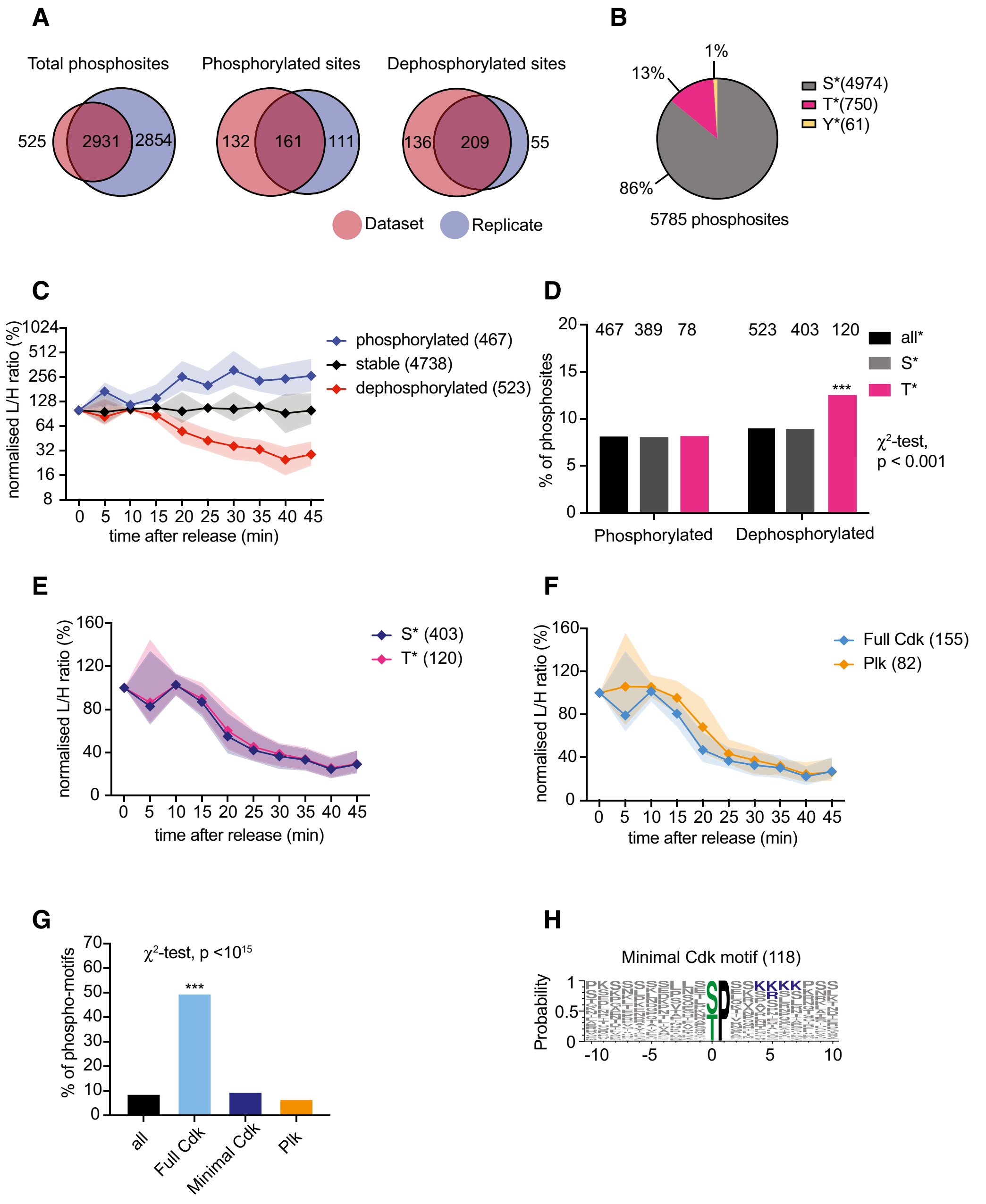

Figure EV3. A repeat SILAC timecourse phosphoproteomics experiment.

A Overlap of phosphosites between our core experimental dataset (red) and the repeat (blue).

B Fraction of serine (S), threonine (T), and tyrosine (Y) phosphosites in the repeat experiment.

C Median intensity profiles and interquartile range of the 467 phosphosites that increased (by at least 50%), the 523 sites that decreased (to under 50%) and the 4,738 sites that remained stable during mitotic progression in the repeat experiment.

D Percentages of phosphorylated and dephosphorylated sites, categorized by phosphoamino acid identity. As in the first analysis, phosphothreonines are significantly preferred dephosphorylation targets (***$P < 0.001$, χ^2-test).

E Median intensity profiles and interquartile range over time of the 403 dephosphorylated serine and 120 threonine sites, confirming their indistinguishable dephosphorylation kinetics.

F Median intensity profiles and interquartile range of the 155 dephosphorylated full Cdk and 82 Polo consensus sites, confirming the later dephosphorylation of Polo sites.

G Percentages of phosphosites in each category that are dephosphorylated, confirming that full Cdk consensus sites are significantly more likely to be dephosphorylated (***$P < 10^{-15}$, χ^2-test).

H All dephosphorylated minimal Cdk motif (S/T)P sites are displayed using Weblogo 3, confirming that these often include an additional positive charge at a downstream position.

© 2018 The Authors

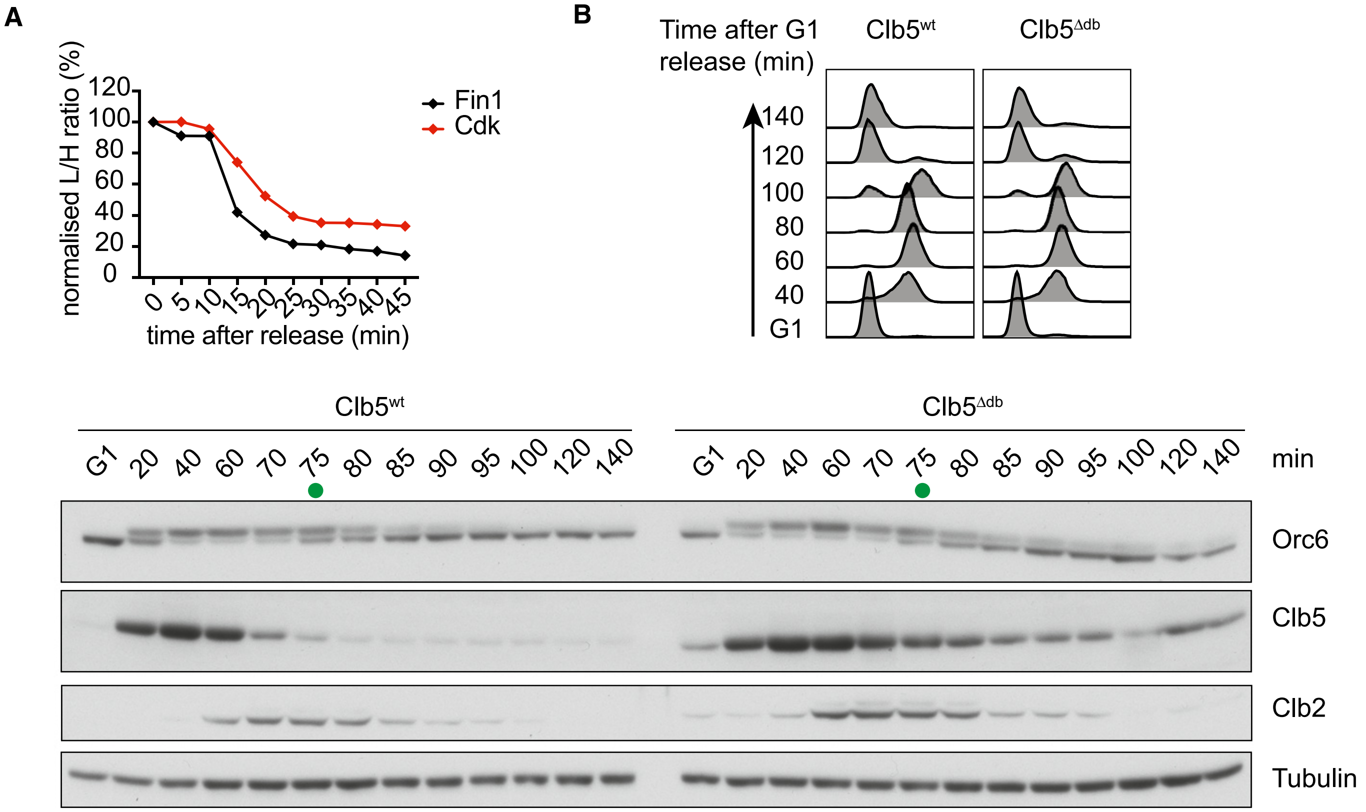

Figure EV4. Clb5 degradation does not impact on Orc6 dephosphorylation.

A Fin1 is dephosphorylated early. The average intensity profile of Fin1 phosphosites and that of all Cdk consensus sites were plotted over time.

B FACS analysis of DNA content confirms α-factor-induced cell cycle arrest in G1, followed by synchronous progression through one cell cycle before re-arrest in the following G1. Cells expressed wild-type Clb5 or Clb5$^{\Delta db}$. Western blotting revealed the Orc6 mobility shift characteristic for its dephosphorylation. Clb5 and Clb2 levels were also analyzed. Tubulin served as a loading control. Green dots indicate the midpoint of Orc6 dephosphorylation.

© 2018 The Authors

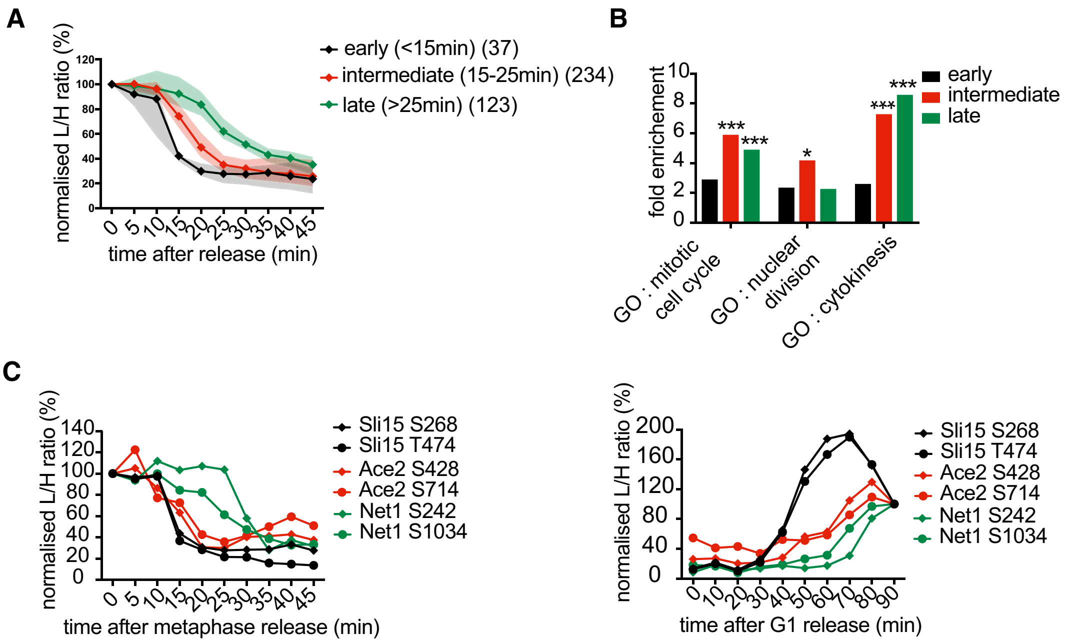

Figure EV5. Classification of early, intermediate, and late substrate dephosphorylation.

A All dephosphorylated sites were categorized by the time when they fell below 50%, early (5–15 min, black), intermediate (20–30 min, red), and late (35 min or later, green). The median intensity profiles and interquartile ranges, as well as the number of phosphosites in each category, are shown.

B Enrichment analysis of gene ontologies (GO) among proteins carrying early, intermediate, and late dephosphorylated sites (**P* < 0.05, ****P* < 0.0001, PANTHER overrepresentation test).

C Phosphosite changes on three example proteins during mitotic exit and during progression from G1 to mitosis. Sli15, Ace2, and Net1 are representatives of the early, intermediate, and late categories, respectively.

 © 2018 The Authors

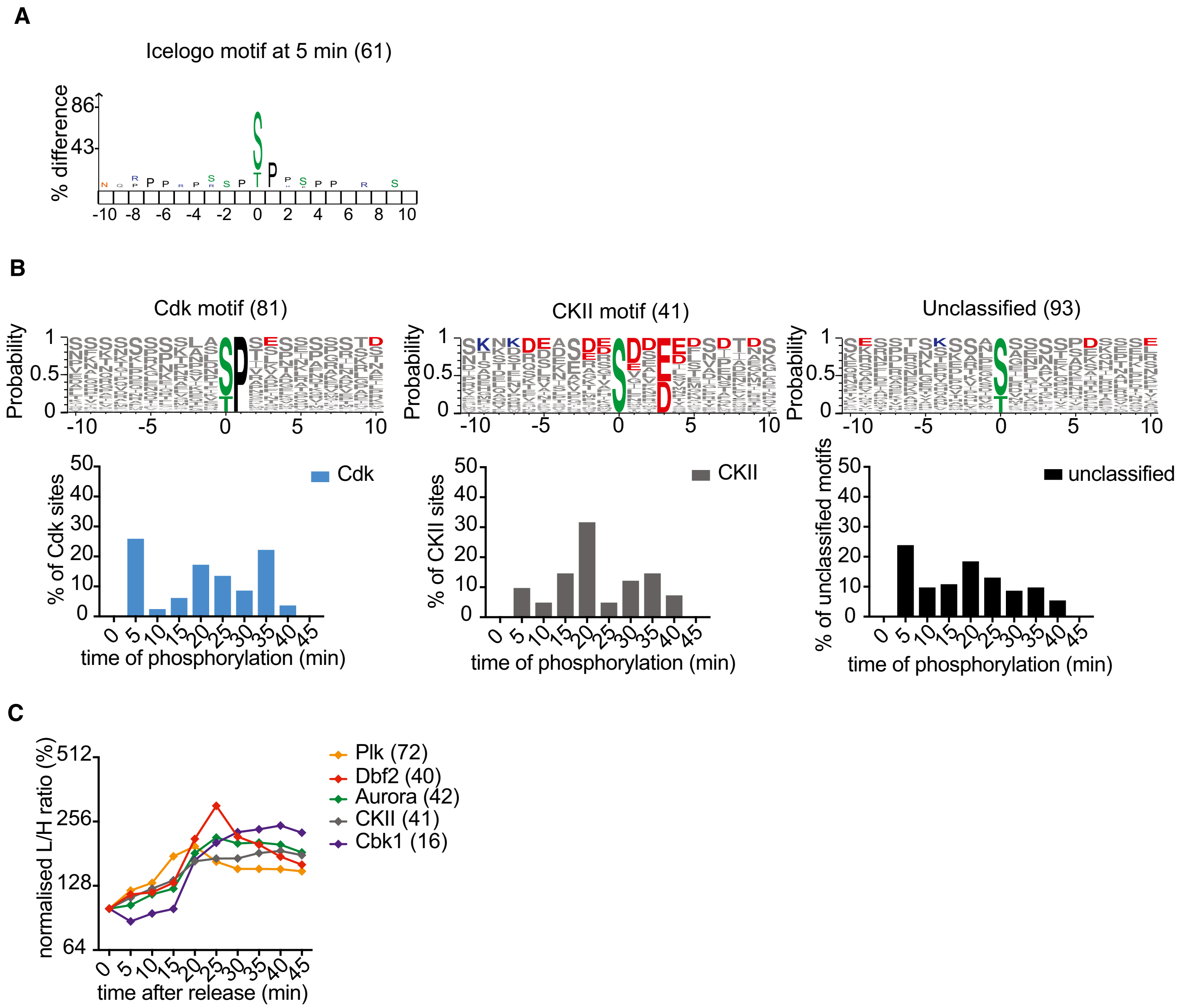

Figure EV6. Motif analysis of mitotic exit phosphorylation.

A IceLogo motif analysis of phosphorylated sites at 5 min.

B Phosphosites that were not covered by motifs analyzed in Fig 6B were divided into those that adhered to a Cdk, or Casein kinase II (CKII) motif, and those that remained unclassified. Surrounding sequences are presented using Weblogo 3 to reveal additional features as above. The number of phosphosites in each category is indicated. Underneath each logo, the distribution of phosphorylation timings of sites in each category is plotted over time.

C Average intensity profiles of phosphosites adhering to the indicated kinase consensus motifs were plotted over the course of mitotic exit.

© 2018 The Authors
