# Supplementary figures and images for: Phosphoproteome dynamics during mitotic exit in budding yeast
Source: EMBO J. 2018 Apr 12;37(10):e98745. doi: 10.15252/embj.201798745 (PMC5978319; doi:10.15252/embj.201798745)

Sic1

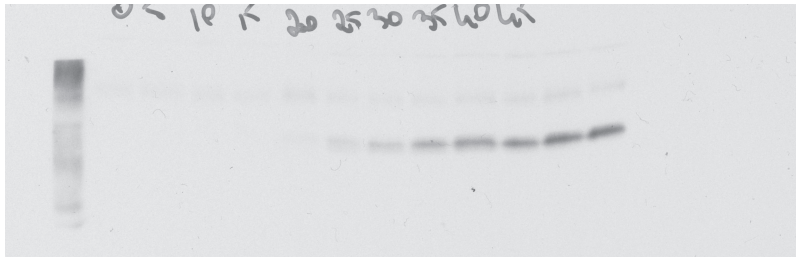

tubulin

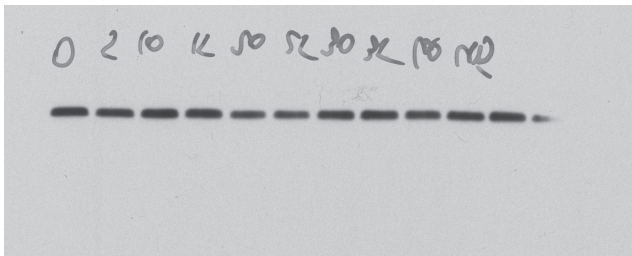

Clb2

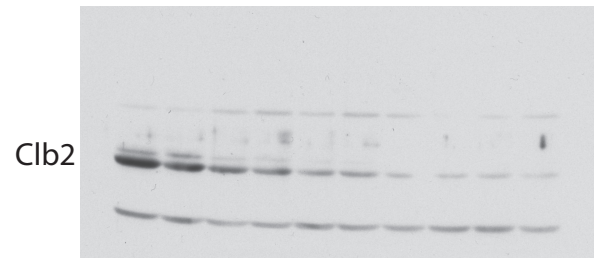

Clb5

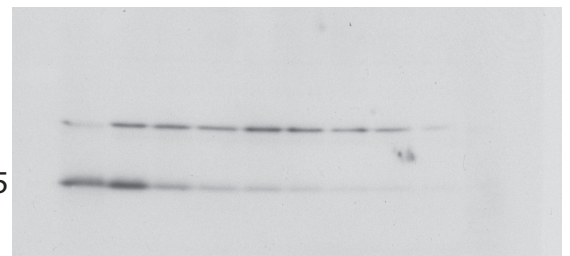

orc6

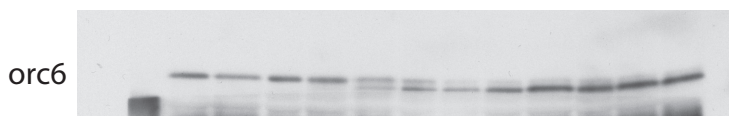

Clb5

Supplement: Supplementary file 8 — Source Data for Figure 1 [file EMBJ-37-e98745-s007.pdf]
